# Prevalence and Associated Risk Factors of Dyslexic Children in a Middle-Sized City of China: A Cross-Sectional Study

**DOI:** 10.1371/journal.pone.0056688

**Published:** 2013-02-15

**Authors:** Zhao Sun, Li Zou, Jiajia Zhang, Shengnan Mo, Shanshan Shao, Rong Zhong, Juntao Ke, Xuzai Lu, Xiaoping Miao, Ranran Song

**Affiliations:** 1 Department of Maternal and Child Health, School of Public Health, Tongji Medical College, Huazhong University of Science and Technology, Wuhan, China; 2 Department of Epidemiology and Biostatistics and MOE Key Lab of Environment and Health, School of Public Health, Tongji Medical College, Huazhong University of Science and Technology, Wuhan, China; 3 Department of Epidemiology and Biostatistics, Arnold School of Public Health, University of South Carolina, Columbia, South Carolina, United States of America; 4 Office of Administration, Renmin Hospital of Wuhan University, Wuhan, China; National Taiwan University Hospital, Taiwan

## Abstract

**Background:**

There are many discussions about dyslexia based on studies conducted in western countries, and some risk factors to dyslexia, such as gender and home literacy environment, have been widely accepted based on these studies. However, to our knowledge, there are few studies focusing on the risk factors of dyslexia in China. Therefore, the aim of our study was to investigate the prevalence of dyslexia and its potential risk factors.

**Methods:**

A cross-sectional study was conducted in Qianjiang, a city in Hubei province, China. Two stages sampling strategy was applied to randomly selected 5 districts and 9 primary schools in Qianjiang. In total, 6,350 students participated in this study and there were 5,063 valid student questionnaires obtained for the final analyses. Additional questionnaires (such as Dyslexia Checklist for Chinese Children and Pupil Rating Scale) were used to identify dyslexic children. The chi-square test and multivariate logistic regression were employed to reveal the potential risk factors to dyslexia.

**Results:**

Our study revealed that the prevalence of dyslexia was 3.9% in Qianjiang city, which is a middle-sized city in China. Among dyslexic children, the gender ratio (boys to girls) was nearly 3∶1. According to the *P*-value in the multivariate logistic regression, the gender (*P*<0.01), mother's education level (*P*<0.01), and learning habits (*P*<0.01) (active learning, scheduled reading time) were associated with dyslexia.

**Conclusion:**

The prevalence rate of dyslexic children in middle-sized cities is 3.9%. The potential risk factors of dyslexic children revealed in this study will have a great impact on detecting and treating dyslexic children in China as early as possible, although more studies are still needed to further investigate the risk factors of dyslexic children in China.

## Introduction

Reading is one of the most important approaches to acquire information in modern society. Developmental dyslexia is defined as a specific and significant impairment in reading ability that cannot be explained by deficits in intelligence, learning opportunity, motivation or sensory acuity [1] and it can occur in areas of basic reading skills, written expression, listening and speaking. It has great wide effect on children involving aspects such as education, career, communication and even health. Some studies demonstrated that the children or adolescents with reading disabilities reported more depress mood, scored higher on the measures of anxiety symptoms and more somatic complaints than youth without reading problems [2], [3], [4]. Other studies showed that the adolescents with dyslexia had higher rates of externalizing behavior problem, aggressive and delinquent behaviors compared with their peers who were normal readers [3], [5], [6]. The importance of research in dyslexia has been well known by educational, medical and social researchers.

Dyslexia is a vital and controversial issue around the world. In western countries, dyslexia has been studied widely during the last several decades. It is agreed that dyslexia is estimated to have a prevalence of 5% to 17% among school-age children [7]. Recently, one population-based birth cohort showed cumulative incidence rates of dyslexia varied from 5.3% to 11.8% depending on the formula used [8]. Whether the boys are more likely to be affected than girls is still in debate [8], [9], [10]. A longitudinal study found no significant difference in the prevalence of dyslexia in research-identified boys compared with research-identified girls. In contrast, they found more dyslexic boys among school-based samples [11]. Besides gender, other risk factors, such as parental education, home literacy, family history, have been investigated. Anne's study showed important differences between adolescents with and without dyslexia relating to psychosocial variables [12]. Moreover, Reanto's study demonstrated associations between dyslexia and gender, season of birth, and age at school entry in a large clinical sample of Italian children [13]. Research on dyslexia developed early and comprehensively in western countries.

However, the knowledge of Chinese dyslexia is limited compared with that in alphabetic language. It is worth pointing out that the results of dyslexia with alphabetic language cannot be generalized to the Chinese dyslexia, because the risk factors associated to dyslexia may differ across cultures and languages. Stevenson et al. found that dyslexia existed among Chinese, Japanese as well as American children [14]. It had been the time that people came to realize the issue existing in Chinese. There are some researches which focused on the cognitive profiles of Chinese dyslexic children in Hong Kong [15], [16], [17], [18], [19], and the similar work has been done in mainland China [20], [21]. Researchers highlighted the importance of morphological awareness for learning to read Chinese and revealed that it might be a core theoretical construct. Although Chinese dyslexia have received more attentions than before, particularly in the brain function using brain imaging techniques [22], [23], [24] which advance in neurology, genetics and psychology, there are still gaps in epidemiological studies in China. Compared with the abundant studies on potential environmental factors of dyslexia and epidemiological survey in alphabetic language countries, research like present study in China is rare. To address the gap, we explored the prevalence rate of dyslexia and possible risk factors, including those related to school and family environment. The Dyslexia Checklist for Chinese Children (DCCC) based on the definition and diagnostic criteria of dyslexia in the Diagnostic and Statistical Manual of Mental Disorders fourth Edition (DSM-IV) was used to diagnose Chinese dyslexic children. This study aims to investigate the prevalence rate of dyslexic children in a middle-sized city and further our understanding of dyslexia which may be influenced by gender, nurture-ways from parents and home literacy environment.

## Methods

### Ethics statement

Written informed consents were obtained from all participants and the next of kin, caretakers, or guardians on the behalf of the participants involved in the study. Besides, the study was approved by the Ethical Committee of Medical Association of Tongji Medical College, Huazhong University of Science and Technology.

### Study design and participants

This study was conducted in Qianjiang, a city located in the southeast of Hubei province, China, which has a population of 1,030,000 with the 2,004 square kilometers [25]. In 2011, there are about 37,927 students in the primary schools. From China census data (2011) [26], there are 657 cities in total China, and among them there are 370 (56.32%) cities defined as the middle-sized (country-level) cities based on the populations and economic status. Qianjiang is one of the 370 middle-sized cities in China, and therefore, results of current study can be generalized to other middle-sized cities. Since there are 22 districts in Qianjiang city and many primary schools in each district, we applied the two-stage sampling to randomly select 5 districts from 22 districts and 9 primary schools from these 5 districts.

All students of third- to sixth-grade in the selected schools were included. The child with dyslexia was recognized based on the following criteria: (a) The score of Dyslexia Checklist for Chinese Children (DCCC) was 2 standard deviations higher than the mean score. The DCCC is an ideal rating scale for Chinese dyslexia with well-established reliability and validity. It consists of 58 items which applied five degrees ranging in scale from 1 to 5 and requires parents to fill out each item according to his/her child's daily behavior. The higher score of DCCC corresponds to the worse reading ability. Referring to our norm of DCCC, the mean score and standard deviation were 105.7 and 33.0 respectively. (b) The score of Pupil Rating Scale Revised Screening for Learning Disabilities (PRS) was lower than 65 points. The PRS is a convenient tool widely used to undertake a diagnose of learning disability in China, containing a total of 24 items divided into five scopes such as listening comprehension, time and spatial judgments, social behavior, motion ability, memory and language ability. The head teacher was demanded to complete the scale depending on the students' performance at school. (c) Chinese language test was below the tenth percentile among all children in the same grade. (d) The intelligence quotient was above 80 assessed by Combined Raven's Test. (e) The child was not suffered from visual and auditory disorders or psychiatric diseases.

### Data collection

Before data collection, we got the permission from all selected schools and informed consents from students and their parents or the next of kin, caretakers, or guardians on the behalf of participants who were invited to participant in the study.

The study was conducted between October 1 and October 31, 2011 by 2 researchers and 5 students with master's degree who are acquaint with the manual of each scale and experienced in epidemical survey. After explanation of our study aims by investigators, the PRS and DCCC scales were respectively completed by the head teachers and guardians depending on the written instruction of the scales. According to the criteria in our study, IQ≥80 was used as an exclusion rule for the subjects, which could be obtained from their medical history in schools. That is, all the subjects enrolled in this study had normal IQ (≥80) by Raven's test. The average score of two unified exams was applied to assess the Chinese language capacity of each student. Besides the scales, we required parents to complete a questionnaire containing three aspects: general information, children's reading habits and home literacy environment. Briefly, general information was collected including children's age, gender, height and weight, medical history, parental education, occupation and family economic status. With regard to children's reading habits, the items about scheduled time on watching TV and surfing internet, whether the children conducting activity reading, whether the children having scheduled reading time were established. Home reading environment was reflected by many items, such as the parents' attitude toward extracurricular reading, how much money spent on the extracurricular books, the frequency of buying books for children, whether the parents telling stories to their children from early childhood, whether the parents having a reading routine, whether the parents buying the books which their children favor.

### Statistical analysis

A descriptive analysis was conducted using mean ± standard deviation (SD) for quantitative variables and frequencies for qualitative variables, respectively. Differences of quantitative or qualitative variables between dyslexic and non-dyslexic children were examined by *t* test or *χ^2^* test. The variables which were statistical significance in *t* test or *χ^2^* test were included in a multivariate logistic regression model. All *P* values were two-tailed with a significant level at 0.05 and all statistical analyses were carried out in SPSS 12.0.

## Results

### Participant characteristics

Totally, there are 6350 students from grade 3 to grade 6 in the selected schools, of which 5536 students returned their questionnaires. However, only 5063 questionnaires were completed, with a response rate of 91.5%. The descriptive statistics of the participants in our study were shown in Table 1. Of the 5063 participants, 2581 (51%) were boys and 2482 (49%) were girls. Their age ranged from 7 to 13 years, with a mean age of 9.4 years (SD = 1.2). With respect to the economic status, 946 (18.7%), 1638 (32.4%), 1909 (37.7%) and 570 (11.3%) families had income of >3000 RMB, 2000–3000 RMB, 1000–2000 RMB, <1000 RMB per month, respectively. Regarding parents' occupation, 25% fathers were professional technical staff and 26.9% mothers were classified as inconvenience, which meant that they had no jobs. According to fathers' education level, 10.1% of fathers had college diploma or above, 18.3% were junior college, 39.8% only completed senior high school or equal, and 31.9% finished junior high school or below. There were 41.1% of mothers with education of junior high school or below, which was the largest proportion among four levels. Mother with college diploma or above and junior college were 6.6% and 14.7% respectively (Table 1).

**Table 1:** Descriptive statistics of the participants (N = 5063).

Variables	N	%
**Dependent variable: dyslexia**		
Non-dyslexia	4868	96.1
Dyslexia	195	3.9
**Gender**		
Boy	2581	51.0
Girl	2482	49.0
**Age(years), Mean ± SD**	9.4±1.2	
**Grade**		
Grade three	1020	20.1
Grade four	1205	23.8
Grade five	1355	26.8
Grade six	1483	29.3
**Variables of socioeconomic status, SES**		
Income of family per month (RMB)		
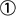 More than 3000 Yuan	946	18.7
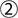 Between 2000 and 3000 Yuan	1638	32.4
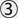 Between 1000 and 2000 Yuan	1909	37.7
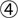 Less than 1000 Yuan	570	11.3
Occupation of father		
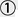 Professional technical staff	1268	25.0
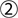 Principal of institution and government	384	7.6
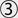 The office staff	360	7.1
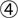 Business staff	649	12.8
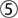 Service staff	360	7.1
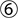 Farming, forestry, fishery worker	407	8.0
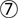 Production worker, transport worker and other related occupations	752	14.9
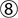 Classification of inconvenience	883	17.4
Occupation of mother		
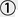 Professional technical staff	611	12.1
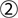 Principal of institution and government	153	3.0
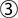 The office staff	350	6.9
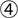 Business staff	767	15.1
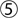 Service staff	873	17.2
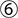 Farming, forestry, fishery worker	404	8.0
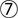 Production worker, transport worker and other related occupations	543	10.7
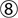 Classification of inconvenience	1362	26.9
Father's education level		
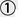 Junior high school or below	1615	31.9
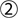 Senior high school or equivalency	2014	39.8
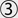 Junior college	925	18.3
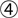 College diploma or above	509	10.1
Mother's education level		
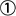 Junior high school or below	2079	41.1
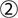 Senior high school or equivalency	1906	37.6
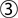 Junior college	743	14.7
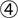 College diploma or above	335	6.6

### The prevalence rate of Chinese dyslexia in Qianjiang city

**Figure 1:**
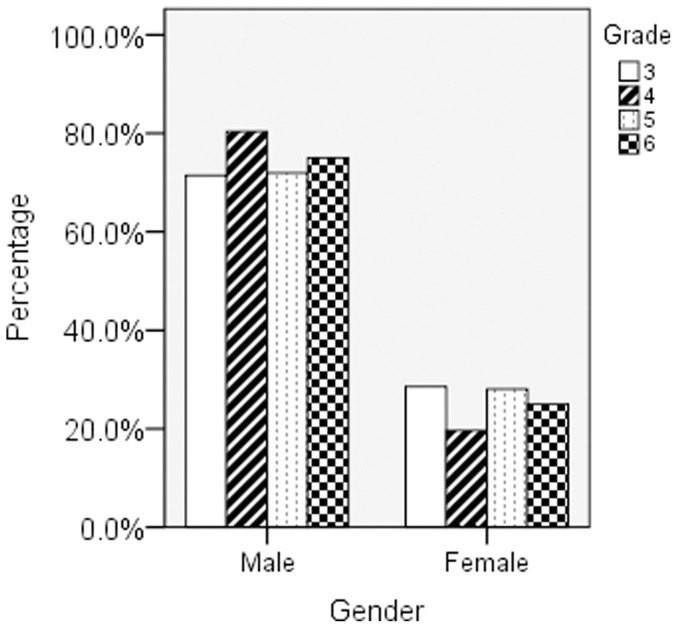
The distribution of dyslexia in grade and gender.

Among total 5063 participants, there were 195 individuals who suffered from dyslexia. The prevalence rate of dyslexia was 3.9% in primary schools in Qianjiang city. Of the 195 children with dyslexia, 146 (74.9%) were boys and 49 (25.1%) were girls, which suggested that the gender ratio of dyslexia was about 3∶1 (boy∶girl). The percentage of dyslexia in each grade was drawn in Figure 1. In 3^rd^ grade, the percentages of boys and girls were 71.4% and 28.6%. Similarly, the percentages were 80.4% and 19.6%, 71.9% and 28.1%, 75% and 25% in 4^th^, 5^th^ and 6^th^ grades respectively.

### The prevalence rate of dyslexia in different grades

As shown in Table 2, the prevalence rates of dyslexia from 3^rd^ to 6^th^ grades were 4.1%, 4.6%, 4.2% and 2.7% separately. There were significant differences on prevalence rates across four grades (*P*<0.05). According to the pair-wise comparisons, significant differences of prevalence rates only existed between 3^rd^ and 6^th^, 4^th^ and 6^th^, 5^th^ and 6^th^ grades (*P*<0.05).

**Table 2:** The prevalence rate of dyslexia in different grades^a^.

Subjects	Grade	Dyslexia (n)	Non-dyslexia (n)	Prevalence rate (%)	*x^2^*	*P*
All grades					8.053	0.045
	3	42	978	4.1		
	4	56	1149	4.6		
	5	57	1298	4.2		
	6	40	1443	2.7		

a There was significant differences between 3^rd^ and 6^th^ grades (*P* = 0.049), 4^th^ and 6^th^ grades (*P* = 0.007), 5^th^ and 6^th^ grades (*P* = 0.027) in prevalence rates separately and no significant differences between 3^rd^ and 4^th^ grades (*P* = 0.544), 3^rd^ and 5^th^ grades (*P* = 0.914), 4^th^ and 5^th^ grades (*P* = 0.588).

### Factors associated with Chinese dyslexia

Table 3 shows the potential risk factors of dyslexia based on the chi-square test. There was significant difference in gender between two groups (*P*<0.05). In dyslexic group, more boys (146, 74.9%) suffered from dyslexia than girls (49, 25.1%), while the number of boys and girls were similar in non-dyslexic group. The average age was 9.35 years for the dyslexia, and 9.39 years for non-dyslexia. The percentages of different economic levels in dyslexia group from high to low levels were 14.9%, 29.2%, 43.1%, 12.8% separately, which were similar to the percentages of non-dyslexia (18.8%, 32.5%, 37.5%, and 11.2%). There was no statistical significance in age and income of family between the two groups (*P*>0.05). Parents' educational level was significantly lower among the dyslexic children than that in non-dyslexia group (*P*<0.05). In dyslexic group, fewer father received education of junior college (25, 12.8%) or above college (3, 1.5%), and fewer mother had attended the junior college (14, 7.2%) or above college (1, 0.5%) when compared with non-dyslexia group.

**Table 3:** Analytical statistics for the dyslexia and non-dyslexia groups.

Variables	Low grade				High grade				All subjects			
	Dyslexia (n = 98)	Non-dyslexia (n = 2127)	*x^2^*	*P*	Dyslexia (n = 97)	Non-dyslexia (n = 2741)	*x^2^*	*P*	Dyslexia (n = 195)	Non-dyslexia (n = 4868)	*x^2^*	*P*
	n(%)	n(%)			n(%)	n(%)			n(%)	n(%)		
**General information**												
Gender			28.7	<0.05			18.6	<0.05			46.3	<0.05
Male	75 (77.0)	1039 (49.0)			71 (73.2)	1396 (50.9)			146 (74.9)	2435 (50.0)		
Female	23 (23.0)	1088 (51.0)			26 (26.8)	1345 (49.1)			49 (25.1)	2433 (50.0)		
Age^a^	8.4±0.7	8.3±0.7	−1.8	>0.05	10.2±0.8	10.2±0.7	−0.4	>0.05	9.35±1.2	9.39±1.2	0.5	>0.05
BMI^b^	16.8±2.8	16.8±4.2	−0.1	>0.05	17.1±3.5	17.7±3.0	−1.8	>0.05	17.25±2.9	16.94±3.8	−1.1	>0.05
Income of family per month			1.0	>0.05			4.3	>0.05			4.2	>0.05
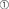 More than 3000 Yuan	15 (15.3)	387 (18.2)			14 (14.4)	530 (19.3)			29 (14.9)	917 (18.8)		
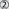 Between 2000 and 3000 Yuan	31 (31.6)	696 (32.7)			26 (26.8)	885 (32.3)			57 (29.2)	1581 (32.5)		
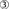 Between 1000 and 2000 Yuan	40 (40.8)	832 (39.1)			44 (45.4)	993 (36.2)			84 (43.1)	1825 (37.5)		
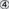 Less than 1000 Yuan	12 (12.2)	212 (10.0)			13 (13.4)	333 (12.1)			25 (12.8)	545 (11.2)		
Father's education level			9.6	<0.05			16.5	<0.05			25.2	<0.05
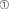 Junior high school or below	39 (39.8)	634 (29.8)			43 (44.3)	899 (32.8)			82 (42.1)	1533 (31.5)		
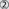 Senior high school or equal	43 (43.9)	883 (41.5)			42 (43.3)	1046 (38.2)			85 (43.6)	1929 (39.6)		
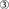 Junior college	13 (13.3)	387 (18.2)			12 (12.4)	513 (18.7)			25 (12.8)	900 (18.5)		
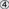 College diploma or above	3 (3.1)	223 (10.5)			0(0)	283 (10.3)			3 (1.5)	506 (10.4)		
Mother's education level			17.2	<0.05			13.3	<0.05			29.3	<0.05
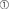 Junior high school or below	52 (53.1)	823 (38.7)			56 (57.7)	1148 (41.9)			108 (55.4)	1971 (40.5)		
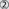 Senior high school or equal	39 (39.8)	802 (37.7)			33 (34.0)	1032 (37.7)			72 (36.9)	1834 (37.7)		
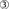 Junior college	7 (7.1)	334 (15.7)			7 (7.2)	395 (14.4)			14 (7.2)	729 (15.0)		
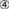 College diploma or above	0 (0)	168 (7.9)			1 (1.0)	166 (6.1)			1 (0.5)	334 (6.9)		
**Learning habits**												
Times of surfing internet per week			4.6	>0.05			0.5	>0.05			3.2	>0.05
1 time or less	82 (83.7)	1608 (75.6)			68 (70.1)	1871 (68.3)			150 (76.9)	3479 (71.5)		
2–4 times	14 (14.3)	413 (19.4)			24 (24.7)	700 (25.5)			38 (19.5)	1113 (22.9)		
5–6 times	2 (2)	54 (2.5)			2 (2.1)	90 (3.3)			4 (2.1)	144 (3)		
7 times or more	2 (2)	52 (2.4)			3 (3.1)	80 (2.9)			3 (1.5)	132 (2.7)		
Hours of surfing per time			1.4	>0.05			0.9	>0.05			1.4	>0.05
Less than an hour	79 (80.6)	1614 (75.9)			60 (61.9)	1722 (62.8)			139 (71.3)	3336 (68.5)		
1–2 hours	16 (16.3)	439 (20.6)			28 (28.9)	829 (30.2)			44 (22.6)	1268 (26.0)		
2–4 hours	3 (3.1)	6 8(3.2)			8 (8.2)	165 (6.0)			11 (5.6)	233 (4.8)		
More than 4 hours	0 (0.0)	6 (0.3)			1 (1.0)	2 5(0.9)			1 (0.5)	31 (0.6)		
Parents' attitude to surfing internet			1.5	>0.05			16.7	<0.05			13.9	<0.05
Ban internet access	23 (23.5)	403 (18.9)			31 (32.0)	459 (16.7)			54 (27.7)	862 (17.7)		
Surf internet only during vacation	28 (28.6)	597 (28.1)			30 (30.9)	861 (31.4)			58 (29.7)	1458 (30.0)		
Surf internet after school	45 (45.9)	1083 (50.9)			34 (35.1)	1362 (49.7)			79 (40.5)	2445 (50.2)		
Up to the child	2 (2.0)	44 (2.1)			2 (2.1)	59 (2.2)			4 (2.1)	103 (2.1)		
Scheduled time on watching TV and surfing internet			7.7	<0.05			16.3	<0.05			20.6	<0.05
Yes, the child can execute	57 (58.2)	1403 (66.0)			41 (42.3)	1677 (61.2)			98 (50.3)	3080 (63.3)		
No	15 (15.3)	385 (18.1)			27 (27.8)	603 (22.0)			42 (21.5)	988 (20.3)		
Yes, the child can't execute	26 (26.5)	339 (15.9)			29 (29.9)	461 (16.8)			55 (28.2)	800 (16.4)		
Hours on watching TV			13.5	<0.05			11.6	<0.05			24.1	<0.05
Less than an hour	41 (41.8)	1044 (49.1)			39 (40.2)	1285 (46.9)			80 (41.0)	2329 (47.8)		
1–2 hours	34 (34.7)	840 (39.5)			34 (35.1)	1104 (40.3)			68 (34.9)	1944 (39.9)		
2–3 hours	14 (14.3)	162 (7.6)			16 (16.5)	233 (8.5)			30 (15.4)	39 (8.1)		
More than 3 hours	9 (9.2)	81 (3.8)			8 (8.2)	119 (4.3)			17 (8.7)	200 (4.1)		
Frequency of parents watching TV with child			4.9	>0.05			1.0	>0.05			4.1	>0.05
Rarely	18 (18.4)	406 (19.1)			22 (22.7)	528 (19.3)			40 (20.5)	934 (19.2)		
Sometimes	68 (69.4)	1274 (59.9)			57 (58.8)	1617 (59.0)			125 (64.1)	2891 (59.4)		
Often	12 (12.2)	447 (21.0)			18 (18.6)	596 (21.7)			30 (15.4)	1043 (21.4)		
Active learning			90.3	<0.05			101.4	<0.05			194.2	<0.05
Yes	8 (8.2)	737 (34.6)			10 (10.3)	1104 (40.3)			18 (9.2)	1841 (37.8)		
Sometimes	50 (51.0)	1162 (54.6)			50 (51.5)	1391 (50.7)			100 (51.3)	2553 (52.4)		
Never	40 (40.8)	228 (10.7)			37 (38.1)	246 (9.0)			77 (39.5)	474 (9.7)		
**Home literacy environment**												
Frequency of parents telling stories			9.1	<0.05			3.2	>0.05			10.1	<0.05
Occasionally	56 (57.1)	999 (47.0)			53 (54.6)	1494 (54.5)			109 (55.9)	2493 (51.2)		
Sometimes	40 (40.8)	896 (42.1)			41 (42.3)	1035 (37.8)			81 (41.5)	1931 (39.7)		
Often	2 (2.0)	232 (10.9)			3 (3.1)	212 (7.7)			5 (2.6)	444 (9.1)		
How about parents encourage the child to read book extra-curricular			8.5	<0.05			4.2	>0.05			12.6	<0.05
Occasionally	16 (16.3)	192 (9.0)			13 (13.4)	246 (9.0)			29 (14.9)	438 (9.0)		
Sometimes	31 (31.6)	564 (26.5)			27 (27.8)	630 (23.0)			58 (29.7)	1194 (24.5)		
Often	51 (52.0)	1371 (64.5)			57 (58.8)	1865 (68.0)			108 (55.4)	3236 (66.5)		
Frequency of parents buying the books child was interested in			11.7	<0.05			7.8	>0.05			18.6	<0.05
Occasionally	26 (26.5)	337 (15.8)			25 (25.8)	489 (17.8)			51 (26.2)	826 (17.0)		
Sometimes	46 (46.9)	920 (43.3)			47 (48.5)	1194 (43.6)			93 (47.7)	2114 (43.4)		
Often	26 (26.5)	870 (40.9)			25 (25.8)	1058 (38.6)			51 (26.2)	1928 (39.6)		
Frequency of parents buying new books			9.5	<0.05			7.2	>0.05			12.8	<0.05
Per week	0 (0.0)	48 (2.3)			2 (2.1)	92 (3.4)			2 (1.0)	140 (2.9)		
Per month	4 (4.1)	258 (12.1)			7 (7.2)	339 (12.4)			11 (5.6)	597 (12.3)		
Per term	13 (13.3)	320 (15.0)			19 (19.6)	414 (15.1)			32 (16.4)	734 (15.1)		
Every year	1 (1.0)	16 (0.8)			3 (3.1)	29 (1.1)			4 (2.1)	45 (0.9)		
Irregularly scheduled, buy when needed	80 (81.6)	1485 (69.8)			66 (68.0)	1867 (68.1)			146 (74.9)	3352 (68.9)		
Frequency of parents' reading			2.9	>0.05			10.9	<0.05			12	<0.05
Every day	18 (18.4)	534 (25.1)			18 (18.6)	751 (27.4)			36 (18.5)	1285 (26.4)		
Every week	50 (51.0)	1059 (49.8)			41 (42.3)	1310 (47.8)			91 (46.7)	2369 (48.7)		
Every month	30 (30.6)	534 (25.1)			38 (39.2)	680 (24.8)			68 (34.9)	1214 (24.9)		
Scheduled reading time			21.6	<0.05			17.8	<0.05			41.2	<0.05
Yes	13 (13.3)	770 (36.2)			26 (26.8)	1331 (48.6)			39 (20.0)	2101 (43.2)		
No	85 (86.7)	1357 (63.8)			71 (73.2)	1410 (51.4)			156 (80.0)	2767 (56.8)		
Expenses of buying books for child every year			8.7	<0.05			10.5	<0.05			16.6	<0.05
Less than 150 Yuan	55 (56.1)	962 (45.2)			59 (60.8)	1234 (45.0)			114 (58.5)	2196 (45.1)		
150–300 Yuan	28 (28.6)	871 (40.9)			28 (28.9)	1032 (37.7)			56 (28.7)	1903 (39.1)		
300–500 Yuan	14(14.3)	225 (10.6)			9 (9.3)	354 (12.9)			23 (11.8)	579 (11.9)		
More than 500 Yuan	1 (1.0)	69 (3.2)			1 (1.0)	121 (4.4)			2 (1.0)	190 (3.9)		

a Age was represented as mean ± standard deviation, *P* value was calculated by the *t* test.

b BMI was represented as mean ± standard deviation, *P* value was calculated by the *t* test.

There was significant difference in parents' attitude regarding to surfing internet between two groups (*P*<0.05). More parents (54, 27.7%) chose to prohibit their children using internet in dyslexia group than those (862, 17.7%) in non-dyslexia group. Hours on watching TV were longer among the dyslexic children than among the non-dyslexia children (*P*<0.05). Among the dyslexic children, 15.4% of children watched TV 2–3 hours every day and 8.7% watched TV more than 3 hours, while they were 8.1% and 4.1% for the non-dyslexia, respectively. The dyslexic children were lack of active learning habit (*P*<0.05). In dyslexia group, fewer children had habit of active learning (18, 9.2%) and more children never learn actively (77, 39.5%) than those in non-dyslexic group.

### Home literacy environment

There was significant association between dyslexia and home literacy environment (*P*<0.05). There were lower frequencies in parents' telling stories (5, 2.6%), encouraging the child to read extra-curricular books (108, 55.4%), buying the books which child was interested in in dyslexia group (51, 26.2%) compared with the non-dyslexia group. There were only 18.5% parents reading every day, which was lower than that in non-dyslexia group (1285, 26.4%). With regard to the children' scheduled reading time, there was a large percentage of children without fixed reading time in dyslexia group (156, 80%), whereas only 6.8% (2767) children had not fixed reading time in non-dyslexia group. Furthermore, the expenses of books varied. There were more parents spending money less than150 RMB on books every year for the dyslexic children (114, 58.5%) than those in non-dyslexia group (2196, 45.1%), and fewer parents (2, 1%) paid more than 500 RMB for books per year in dyslexia group than those in non-dyslexia group (190, 3.9%).

### Multivariate logistic regression

There were significant associations between dyslexia and gender, parental education level, attitude to using internet, scheduled time on TV and internet, active learning and home literacy environment (Table 3). To estimate the effect sizes of these possible risk factors, we conducted a multivariate logistic regression analysis. Table 4 shows ORs and 95% CIs for the potential risk factors. From table 4, we could see that there was a significant difference in gender between two groups and the OR in boys who suffered from dyslexia was 2.3 times as high as that in girls. Low educational level of mothers was a risk factor for dyslexia (junior high school or below: OR = 12.1, 95% CI: 1.7–87.3; senior high school or equivalency: OR = 9.3, 95% CI: 1.3–67.4) compared with mothers with higher education level. Active learning had positive effect on dyslexia with OR being 0.1 and 0.3 among children who always or sometimes had active learning habit. For children with scheduled reading time, the OR to be dyslexic was 0.5 times as high as those children without scheduled reading time. Therefore, we concluded that dyslexia was significantly associated with child's gender, mother's education level, active learning, and scheduled reading time.

**Table 4:** Multivariate logistic regression analysis of associated factors for dyslexia.

Subjects	Variables	OR (95%CI)	*P*
Low-grade			
	**Gender**		
	Female	1	
	Male	2.7 (1.7–4.4)	<0.001
	**Active learning**		
	Never	1	
	Yes	0.1 (0.0–0.2)	<0.001
	Sometimes	0.3 (0.2–0.5)	<0.001
High-grade			
	**Gender**		
	Female	1	
	Male	2.0 (1.2–3.1)	0.004
	**Active learning**		
	Never	1	
	Yes	0.1 (0.0–0.1)	<0.001
	Sometimes	0.3 (0.2–0.4)	<0.001
All subjects			
	**Gender**		
	Female	1	
	Male	2.3 (1.6–3.2)	<0.001
	**Mother's education level**		
	College diploma or above	1	
	Junior college	5.0 (0.7–38.5)	0.122
	Senior high school or equivalency	9.3 (1.3–67.4)	0.028
	Junior high school or below	12.1 (1.6–87.3)	0.014
	**Active learning**		
	Never	1	
	Yes	0.1 (0.1–0.2)	<0.001
	Sometimes	0.3 (0.2–0.4)	<0.001
	**Schedule reading time**		
	No	1	
	Yes	0.5 (0.4–0.8)	0.002

OR: odds ratio, CI: confidence interval.

### Risk factors associated with dyslexia for low and high grades students

We divided the subjects into low (3–4 grades) and high grades (5–6 grades). Based on Table 3, common risk factors for both grades include gender, parents' education level, scheduled time on watching TV and surfing internet, whether the children conducting active learning, whether the children having scheduled reading time, and expenses of buying books for children. There were several risk factors specific to low and high grades. For example, the factors including the frequency of parents telling stories to their children, parents buying extracurricular books for their children and parents buying new books, the parents' attitude toward extracurricular reading were significant associated with dyslexia in low grades. While parents' attitude toward surfing internet, and whether parents having a reading routine were found to be risk factors for high grades. We also conducted the multivariate logistic regression, and found that gender and whether the children conducting activity reading were related to dyslexia risk for both grades (Table 4).

## Discussion

To our knowledge, the current study is one of the first studies focusing on the prevalence and risk factors for Chinese dyslexic children. Not only did we use the DCCC scale to evaluate the dyslexia among children, but also we considered their school and family environment. The main findings from this study were (1) The prevalence of dyslexia was 3.90% in Qianjiang city and the prevalence of dyslexia in boys was higher than that in girls. (2) Learning habits were significantly associated with dyslexia. (3) Home literacy environment could have impact on children's reading abilities.

Depending on the definition of dyslexia and culture differences, the prevalence rate varies. According to a previous study [14], children learning to read Chinese and Japanese suffered from the dyslexia, as well as their peers who learned English. Considering low reading ability together with average IQ, 7.5% children in Taipei met the criteria of dyslexia. If researchers applied the low achievement criteria that the child who fall behind average reading level two standard deviation was regarded as dyslexia [27], 2% of Taipei children [14] were thought to have reading disability. The difference between our study and the one in Taipei may come from the difference of two types of Chinese characters. The simplified Chinese characters are always adopted in mainland, while in Taiwan, the traditional Chinese is widely used [28]. The average number of strokes of 2000 commonly used characters is 11.2 for the traditional script used in Hong Kong and Taiwan and 9.0 for the simplified script used in Mainland China [17]. Therefore, it is harder for children to recognize the traditional characters than the simplified ones. We also noticed that the difference in the learning mechanism. Children learn to read Chinese characters via Pin-Yin (an alphabetic phonetic system) in Mainland China and Zhu-Yin-Fu-Hao (a sub-syllabic phonetic system) in Taiwan. Although both of them come from the same language origin, the difference in phonetic system may affect the prevalence of dyslexia in mainland China.

Later, a similar study was conducted in mainland China, which reported that 4.55% children were subjected to dyslexia. In our study, we also adopted the low achievement criteria. The prevalence of dyslexia in this survey was 3.9%, which was between those of above two studies. The difference may be caused by the diversity in the study subjects. In the above two studies, subjects were students in 5^th^ grade of the primary schools, but the subjects of our study were selected from 3^rd^–6^th^. As early as 3^rd^ grade, children have showed academic difficulties evidently. By the time whether the children suffered from dyslexia or not could be entrenched. Thus, the current results provide a better knowledge of Chinese dyslexia based on populations in the primary schools. So far, the association between prevalence rate and grade is unclear. Theoretically, the prevalence of dyslexia will be lower in lower grade, because many students may manifest the symptoms of dyslexia in higher grade. However, the prevalence of dyslexics in grade 6 was the lowest among 4 grades in this study. It may be explained by a previous study, which demonstrated that the prevalence of dyslexia in higher grade was likely to be lower than the one in lower grade, because the symptoms of some dyslexic children in higher grade would be improved through the systematic reading experience of one or two years [7]. Therefore, more studies are called to address this problem in the future.

The prevalence of Chinese dyslexia is lower than the reported prevalence rate in western countries, which were 5% to 17% [7]. The study by Siok et al. [24] provided an insight into the fundamental path physiology of dyslexia by suggesting that rather than having a universal origin, the biological abnormality of impaired reading depends on culture. Indeed, Chinese belongs to non-alphabetic languages which may result in specificity in dyslexia in contrast with alphabetic languages. The percentage of dyslexia in Chinese is lower than their peers who speak English, but the relative amount of dyslexic children is still high in China. It is necessary to employ an epidemiological investigation in China. Therefore, we need to take great effort to diagnose and treat dyslexic adolescents as early as possible.

In current study, a gender ratio was nearly 3∶1 from boys to girls for dyslexic children. The logistic regression revealed that the risk of boys was 2.3 times as high as girls. A large body of studies has typically reported that there were significantly more boys than girls with dyslexia [29], [30]. Our findings are coincident with previous studies. The possible reasons may be genetic difference, inherited features and development of cognition. Normand reported that a small advantage in language production for girls over boys until 36 months of age [31]. In China, boys are always expected to be proficient at science subjects, while girls are expected to be good at arts. For parents, to some extent, child's gender will influence their attitude and educational ways to child. Our study indicated that boys were more likely to be dyslexic. The gender difference in dyslexia suggested that teaching methods should be considered for boys and girls.

Our results suggested that learning habits and parents' education level were associated with dyslexia. The higher education levels the parents have, the less risk their children are suffered from. And father's higher education always relates to a better social economic status. Furthermore, education and income are the determinants of socioeconomic status which are associated with the development of children language reported by previous research [32]. Maternal characteristics such as verbal ability and personality have been shown to be related to the construction of the child's environment, therefore, influence the child's development [33]. In the previous studies, the conclusion that maternal education had been strongly associated with reading and literacy had been reported, which was confirmed by the results of current study. The traditional idea that man should work outside while woman should do housework at home has not been converted completely in China. Therefore, mother communicates with child more frequently and plays an important role in raising the child. Because of the different cognitive ability, mother with different educational level may create various reading environment. Learning habits including active learning and scheduled reading time were potential factors for dyslexia, which were seldom reported in the previous studies. Child who is active in learning behaves actively during the learning, including asking questions, discussing with teachers or classmates frequently and so on. Dyslexic children usually have difficulty in reading and the failure may decrease the motivation of learning. The interaction between active learning and dyslexia may circulate towards to worse situation. That is, those children will experience more depressed, unhappy, bored feelings to reading. In China, parents usually expect their child to achieve a higher academic goal no matter their ability, and the child endures enormous pressure. For the child with dyslexia, the pressure might be larger which cannot be assessed. The study also showed that scheduled reading time was associated with dyslexia. Motivation is important to learning because of its link to reading practice [34]. As a result, parents' guide and encouragement are crucial to children's reading. In summary, students who cannot read well and read less usually lost practice opportunities, which makes it difficult to acquire average level of reading fluency. Reading experience is the vital access to increase vocabularies, reading comprehension and related skills. Because of limited knowledge and notion, parents often ignore the importance of reading practice. Thus, it is necessary to establish reading time for children.

Home literacy environment was found to associate with dyslexia in our study. When parents tell stories to child and encourage child to read more frequently, their child has less risk of dyslexia. While the parents buy books that child is interested in, their child is less likely to be dyslexia. Fontina L. Rashid indicated that children's home literacy actives were not significantly related to any of their academic abilities, whereas parents' home literacy activities were significantly related to children's passage comprehension and spelling scores [35]. According to our results, parents' reading activities (spending more time to reading with child, more books available at home, encouraging child to read) can help to reduce the risk of dyslexia.

The difference of risk factors associated with dyslexia in low grade and high grade indicated the importance of parents. Children in high grade may face heavy pressure of entering high schools so that their parents pay more attention to their scores instead of offering a good literacy environment. Generally speaking, children in high grade process reading practice mainly in class. As a result, there were no associations between parents' relevant actives (frequency of parents tell stories, how about parents encourage the child to read book extra-curricular, frequency of parents buy the books child interested) and dyslexia. On the other hand, children in low grade will be relaxed and parents' relevant actives may be associated with dyslexia. Therefore, parents' attitude to construct what kind of literacy environment is crucial for their children.

The current study showed special culture, economy and educational system may be associated with dyslexia. Additional studies are still needed to explore issues in debate. However, there are still some shortcomings in present study. The current study was carried out in one city in China, and we could only generalize the prevalence rate to other middle-sized cities similar to Qianjiang city. More different types of cities should be sampled if we want to know the dyslexic prevalence in the whole China in the future studies. The subjects were selected from primary schools, which just reflected the prevalence rate of dyslexia among children from 9–12 years old. To a great extent, the etiology of Chinese dyslexia is still uncovered. The learning habits, home literacy environment and the other factors such as cognitive characters of children will continue to be studied in the future.
